# In Silico Inhibition Studies of Jun-Fos-DNA Complex Formation by Curcumin Derivatives

**DOI:** 10.1155/2012/316972

**Published:** 2012-12-06

**Authors:** Anil Kumar, Utpal Bora

**Affiliations:** ^1^Computational Biology Laboratory, Department of Biotechnology, Indian Institute of Technology Guwahati, Assam, Guwahati 781039, India; ^2^Biotech Hub, Centre for the Environment, Indian Institute of Technology Guwahati, Assam, Guwahati 781039, India

## Abstract

Activator protein-1 (AP1) is a transcription factor that consists of the Jun and Fos family proteins. It regulates gene expression in response to a variety of stimuli and controls cellular processes including proliferation, transformation, inflammation, and innate immune responses. AP1 binds specifically to 12-O-tetradecanoylphorbol-13-acetate (TPA) responsive element 5′-TGAG/CTCA-3′ (AP1 site). It has been found constitutively active in breast, ovarian, cervical, and lung cancers. Numerous studies have shown that inhibition of AP1 could be a promising strategy for cancer therapeutic applications. The present in silico study provides insights into the inhibition of Jun-Fos-DNA complex formation by curcumin derivatives. These derivatives interact with the amino acid residues like Arg155 and Arg158 which play a key role in binding of Jun-Fos complex to DNA (AP1 site). Ala151, Ala275, Leu283, and Ile286 were the residues present at binding site which could contribute to hydrophobic contacts with inhibitor molecules. Curcumin sulphate was predicted to be the most potent inhibitor amongst all the natural curcumin derivatives docked.

## 1. Introduction

Activator protein-1 (AP1) is a transcription factor that consists of either homo- or heterodimers of the Jun and Fos family proteins [1]. It regulates gene expression in response to a variety of stimuli, including environmental stresses, UV radiation, cytokines, and growth factors. AP1 in turn controls a number of cellular processes including proliferation, transformation, inflammation, and innate immune response. The Jun and Fos proteins share similar amino acid sequences that comprise the basic DNA-binding sequence and the adjacent leucine zipper region by which these proteins dimerize [2–4]. The AP1 transcription factor binds specifically to 12-O-tetradecanoylphorbol-13-acetate (TPA) responsive element 5′-TGAG/CTCA-3′ which is commonly referred to as the AP1 site [5, 6]. *C-fos* and *c-jun* genes are autoregulated; the transcription of *c-jun* is stimulated by its own product, and in contrast *c-fos* is negatively autoregulated [7–9]. AP1 has been found constitutively active in many cancers including breast, ovarian, cervical, and lung. Numerous studies have shown that inhibition of AP1 has a profound effect on the behavior of cancer cells and tumors suggesting that AP1 could be a promising target for cancer therapy [10].

Curcumin, a dietary spice derived from the plant Turmeric (*Curcuma longa*), is used as a traditional medicine for inflammatory conditions [11]. Further, curcumin has been reported to have anti-inflammatory, anti-oxidant, and anticancer effects [12–15]. In vivo administration of curcumin was found to reduce the incidence and size of tumors in mice [16–19]. Moreover, curcumin was reported to inhibit proliferation and cell cycle progression in cancer cells [20]. Curcumin suppresses constitutive AP1 activity in HL-60, Raji, and prostate cancer cell lines (LNCaP, PC-3, and DU145) [21–25]. Curcumin was also reported to suppress LPS-induced cyclooxygenase-2 gene expression by inhibiting AP1 DNA binding in BV2 microglial cells [26]. It was confirmed that curcumin directly interacts with Jun-Fos dimer and inhibits its binding to DNA (AP1 site) [27]. Some synthetic curcumin derivatives have been discovered as inhibitors of Jun-Fos-DNA complex formation [28–30]. However, no information on the site of interaction is reported yet. In the present study we investigate the interaction of curcumin derivatives with Jun-Fos complex by molecular docking studies.

## 2. Methodology

**Figure 1:**
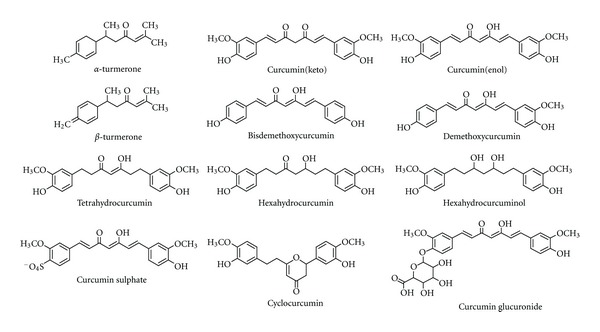
Chemical structures of natural curcumin derivatives.

**Figure 2:**
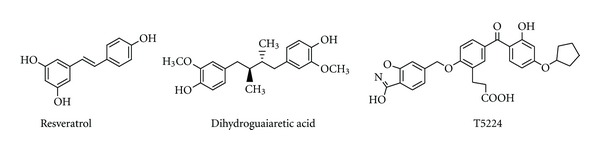
Known inhibitors of Jun-Fos-DNA complex formation used in the study.

To investigate the interaction with Jun-Fos complex, curcumin natural derivatives (Figure 1), synthetic curcumin-based inhibitors (Table 1), and other known inhibitors of Jun-Fos-DNA complex formation (Figure 2) were drawn and 3D optimized using MarvinSketch (Free Academic License) and saved in Protein Data Bank (PDB) file format [31]. These molecules were prepared for molecular docking by merging nonpolar hydrogens, assigning Gastegier charges, and saving them in PDBQT file format using AutoDock Tools (ADT) 1.5.6 [32]. X-ray crystal structure of Jun-Fos-DNA complex (PDB ID: FOS1) was obtained from the Protein Data Bank (http://www.rcsb.org/pdb). For molecular docking DNA and other heteroatoms (water, ions, etc.) were removed using PyMOL 0.99. Gasteiger charges were assigned, and Jun-Fos complex was saved in PDBQT file format using ADT.

Grid and docking parameter files were prepared using ADT, and molecular docking was performed with AutoDock 4.2.1 (Scripps Research Institute, USA) considering all the rotatable bonds of curcumin derivatives as rotatable and Jun-Fos complex as rigid [33]. Grid box size of 90 × 90 × 90 Å with 0.375 Å spacing was selected that include the whole basic DNA-binding sequence and the adjacent leucine zipper region of Jun-Fos complex. Empirical-free energy function and Lamarckian genetic algorithm, with an initial population of 150 randomly placed individuals, a maximum number of 2,500,000 energy evaluations, a mutation rate of 0.02, and a crossover rate of 0.80, were used to perform molecular docking. Hundred independent docking runs were performed for each molecule. Curcumin derivative-Jun-Fos complex for lowest free energy of binding (Δ*G*) confirmation from the largest cluster was written in PDBQT format and converted to PDB file format using UCSF Chimera 1.6.1. Further, these complexes were analyzed using PyMOL 0.99 for possible polar and hydrophobic interactions. All the docking studies were performed at Intel(R) Xeon(R) CPU (3.2 GHz) with Linux-based operating system Fedora 15.

## 3. Results and Discussions

**Figure 3:**
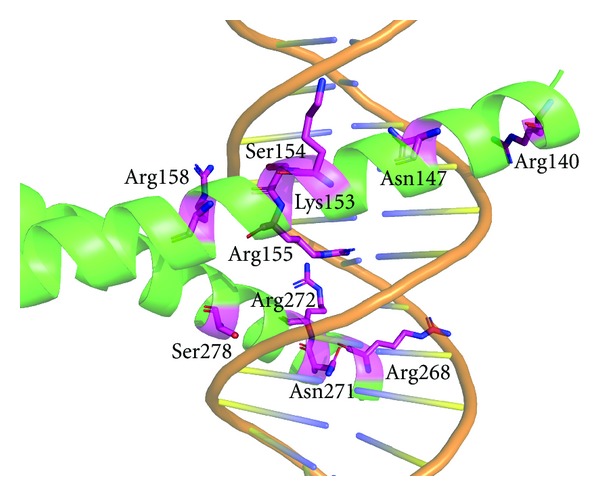
X-ray crystal structure of Jun-Fos-DNA complex (PDB ID: FOS1) showing amino acid residues (magenta) which form hydrogen bond with DNA (AP1 site).

X-ray crystal structure of Jun-Fos-DNA complex shows that Arg140, Asn147, Lys153, Ser154, Arg155, Arg158, Arg268, Asn271, Arg272, and Ser278 are the key residues by which Jun-Fos complex binds to DNA through hydrogen bonding (Figure 3). To predict the interaction of curcumin derivatives with Jun-Fos complex, natural curcumin derivatives and other known inhibitors of Jun-Fos-DNA complex formation were docked over DNA-binding region (DBR) of Jun-Fos complex, and results were summarized in Table 2.

**Figure 4:**
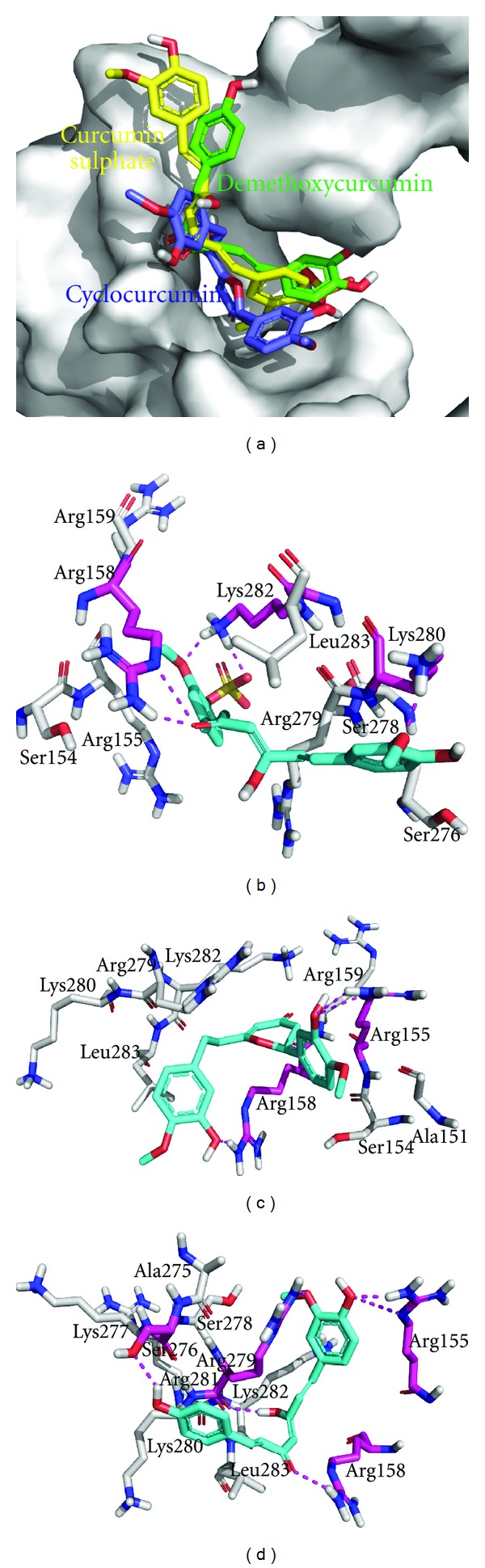
Binding modes of natural curcumin derivatives. (a) Curcumin sulphate (yellow), cyclocurcumin (blue), and demethoxycurcumin (green) docked to DBR of Jun-Fos complex; (b) curcumin sulphate (cyan) showing polar contacts with Arg158, Lys280, and Lys282 (magenta) (c) cyclocurcumin showing polar contacts with Arg155 and Arg158 (magenta); (d) demethoxycurcumin showing polar contacts with Arg155, Arg158, Ser276, and Arg279 (magenta).

Amongst all the natural curcumin derivatives docked to Jun-Fos complex curcumin sulphate bound with Δ*G* of −8.20 kcal/mol and predicted KI of 976.64 nM followed by cyclocurcumin and demethoxycurcumin which bound with Δ*G* of −5.75 and −5.72 kcal/mol and predicted KI of 61.42 and 63.86 *μ*M, respectively (Figure 4(a)). The binding mode of curcumin sulphate depicted that sulphate and nearby methoxy group present at one aromatic ring of the molecule were in polar contact range with Lys282; however methoxy group present at the other side formed polar contact with side chain of Lys280 (Figure 4(b)). Keto group present in the linker region was in polar contact range with side chain of Arg158. The binding mode of cyclocurcumin showed that hydroxyl group present at one aromatic ring of the molecule formed polar contact with side chain of Arg155; however at the other side it formed polar contacts with Arg158 (Figure 4(c)). When demethoxycurcumin docked to Jun-Fos complex, hydroxyl and neighboring methoxy group present at one aromatic ring formed polar contact with side chains of Arg155 and Arg279, respectively, while hydroxyl group present at other side of the molecule formed polar contact with side chain of Ser276 (Figure 4(d)). In the linker region of the molecule keto and hydroxyl groups were in polar contact range with Arg158 and Arg279, respectively. 

**Figure 5:**
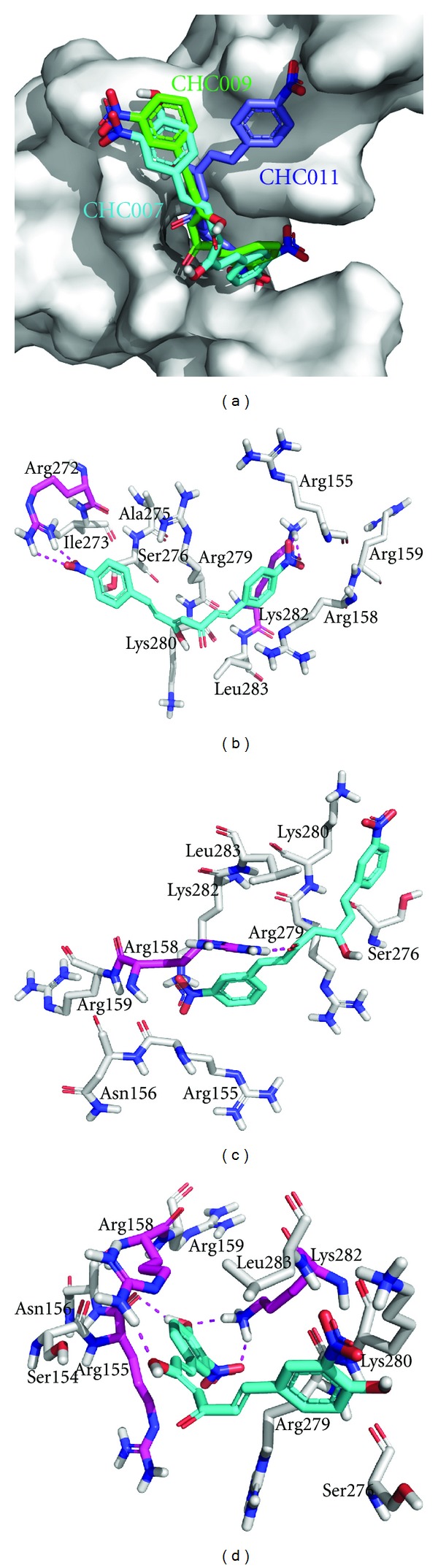
Binding modes of synthetic curcumin-based inhibitors (a) CHC011 (blue), CHC009 (green), and CHC007 (cyan) docked to DBR of Jun-Fos complex; (b) CHC011 (cyan) showing polar contacts with Arg272 and Lys282 (magenta); (c) CHC009 (cyan) showing polar contacts with Arg158 (magenta). (d) CHC007 (cyan) showing polar contacts with Arg155, Arg158, and Lys282 (magenta).

Amongst the synthetic curcumin-based inhibitors CHC011 bound to Jun-Fos complex with Δ*G* of −9.59 kcal/mol and predicted KI of 93.25 nM followed by CHC009 and CHC007 which docked with Δ*G* of −9.52 and −9.15 kcal/mol and predicted KI of 104.26 nM and 196.96 nM, respectively (Figure 5(a)). Similar results were observed in the in vitro studies by Hahm et al. in 2002 [28]. The binding mode studies depicted that –NO_2_ group present at one aromatic ring of the CHC011 molecule formed polar contact with side chain of Arg272 while at the other side of the molecule it interacted with Lys282 (Figure 5(b)). When CHC009 docked to Jun-Fos complex, keto group present in the linker region of the molecule formed polar contact with side chain of Arg158 (Figure 5(c)). Hydroxyl and –NO_2_ group present at one aromatic ring of the CHC007 molecule formed polar contacts with backbone of Arg155 and side chain of Lys282, respectively, while the hydroxyl group present in the linker region of the molecule showed polar contact with side chain of Arg158 (Figure 5(d)). 

**Figure 6:**
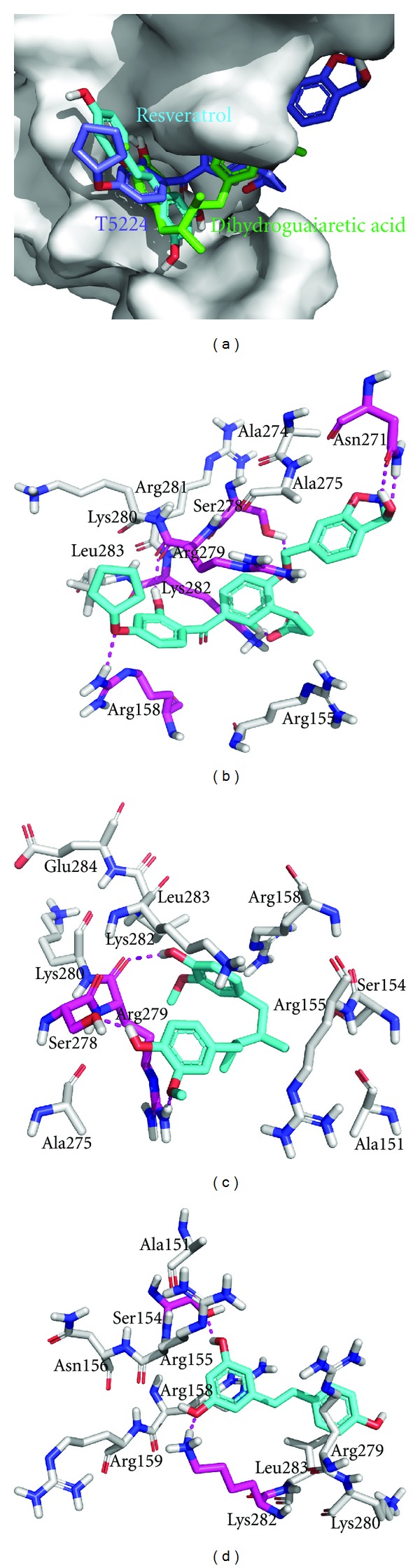
Binding modes of other known inhibitors. (a) T5224 (blue), dihydroguaiaretic acid (green), and resveratrol (cyan) docked to DBR of Jun-Fos complex (b) T5224 (cyan) showing polar contacts with Arg158, Asn271, Ser278, Arg279, and Lys282 (magenta); (c) Dihydroguaiaretic acid (cyan) showing polar contacts with Ser278 and Arg279 (magenta); (d) resveratrol (cyan) showing polar contacts with Ser154 and Lys282 (magenta).

Amongst the other known inhibitors T5224 [3-(5-(4-(cyclopentyloxy)-2-hydroxybenzoyl)-2-((3-hydroxybenzo [d]isoxazol-6-yl)methoxy)phenyl)propanoic acid] bound to Jun-Fos complex with Δ*G* of −9.96 kcal/mol and predicted KI of 49.64 nM followed by dihydroguaiaretic acid and resveratrol which docked with Δ*G* of −4.43 and −4.20 kcal/mol and predicted KI of 569.58 and 829.30 *μ*M, respectively (Figure 6(a)). The binding mode studies of T5224 depicted that oxygen atom of cyclopentyloxy group formed polar contact with side chain of Arg158; however nearby hydroxyl group formed polar contact with Arg279. Hydroxyl group of 3-hydroxybenzo [d]isoxazol-6-yl)methoxy group formed polar contact with Asn271; however oxygen atom of its methoxy group formed polar contact with Ser278. Acid group of the T5224 molecule was in polar contact range with Lys282 (Figure 6(b)). When docked to Jun-Fos complex neighboring hydroxyl and methoxy groups present at one side of the dihydroguaiaretic acid molecule formed polar contacts with Ser278 and Arg279 respectively, whereas the hydroxyl group present at the other side of the molecule formed polar contact with backbone of Arg279 (Figure 6(c)). When docked to Jun-Fos complex neighboring hydroxyl groups attached to one of the aromatic ring of resveratrol molecule formed polar contacts with Ser 154 and side chain of Lys282, respectively (Figure 6(d)).

We observed that curcumin derivatives form polar contacts preferentially with residues like Arg155, Arg158, Lys276, Arg279, Lys280, and Lys282 when docked to DBR of Jun-Fos complex amongst which Arg155 and Arg158 are the key residues by which Jun-Fos complex binds to DNA. The results suggested that interaction of curcumin derivatives with residues like Arg155 and Arg158 could be the possible mechanism by which curcumin derivatives inhibit Jun-Fos-DNA complex formation. Ala151, Ala275, Leu283, and Ile286 were the hydrophobic residues present at binding site contributing to hydrophobic contacts with inhibitor molecules. 

## 4. Conclusions

The present molecular docking study provides insights into the inhibition of Jun-Fos-DNA complex formation by curcumin derivatives. The involvement of residues like Arg155, Arg158, Lys276, Lys280, and Lys282 seems to play a key role in binding of curcumin derivatives to Jun-Fos complex through polar contacts which prevents its binding to DNA (AP1 site). Ala151, Ala275, Leu283 and Ile286 were the important hydrophobic residues present at binding site. Most of the curcumin derivatives were predicted to be more potent than inhibitors like resveratrol and dihydroguaiaretic acid. Curcumin sulphate was predicted to be the most potent inhibitor amongst all the natural curcumin derivatives docked. 

## Figures and Tables

**Table 1:** Synthetic curcumin-based inhibitors of Jun-Fos-DNA complex formation.

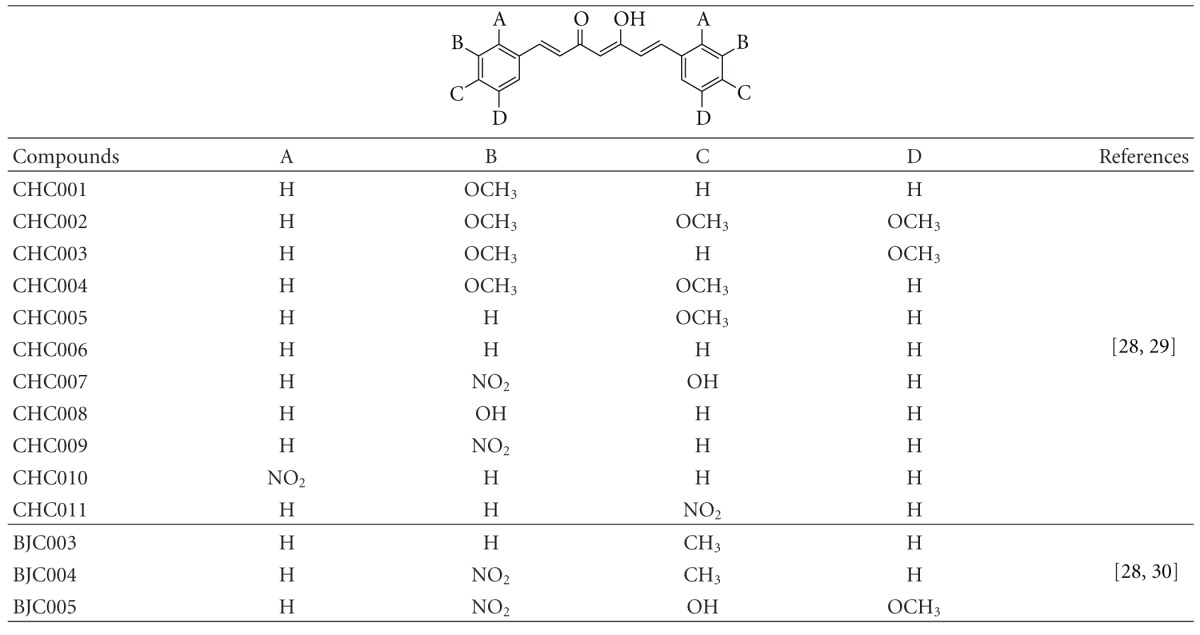

**Table 2:** Free energy of binding (Δ*G*) and predicted inhibition constant (KI) estimated with AutoDock 4.2.1 and interaction of inhibitors with Jun-Fos complex.

Compounds	Δ*G* (kcal/mol)	KI	Putative polar interactions	Hydrophobic residues in 5 Å region
T5224^*ψ*^	−9.96	49.64 nM	Arg158, Asn271, Ser278, Arg279, Lys282	Ala274, Ala275, Leu283
CHC011∗	−9.59	−93.25 nM	Arg272, Lys282	Ile273, Ala275, Leu283
CHC009∗	−9.52	104.26 nM	Arg158	Leu283
CHC007∗	−9.15	196.96 nM	Arg155, Arg158, Lys282	Leu283
BJC004∗	−9.12	207.86 nM	Lys153	Ala150, Ala151
BJC005∗	−8.94	277.86 nM	Arg155, Arg158, Lys280, Lys282	Ala275, Leu283, Ile286
Curcumin sulphate	−8.20	976.64 nM	Arg158, Lys280, Lys282	Leu283
CHC010∗	−6.73	11.59 *μ*M	Ser278, Arg279	Ala274, Ala275
CHC008∗	−5.86	50.65 *μ*M	Arg155, Arg158, Ser-276, Lys282	Leu283
Cyclocurcumin	−5.75	61.42 *μ*M	Arg155, Arg158	Ala151, Leu283
CHC003∗	−5.73	62.98 *μ*M	Arg158, Arg279, Lys280, Lys282	Ala275, Leu283
Demethoxycurcumin	−5.72	63.86 *μ*M	Arg155, Arg158, Ser276, Arg279	Ala275, Leu283
BJC003∗	−5.69	67.22 *μ*M	Arg158, Arg279	Leu283
CHC004∗	−5.66	71.36 *μ*M	Arg158, Lys280, Lys282	Leu283
CHC006∗	−5.45	101.79 *μ*M	Arg158, Arg279	Leu283
CHC002∗	−5.32	125.57 *μ*M	Arg155, Arg158, Arg279, Lys280, Lys282	Ala275, Leu283
Bisdemethoxycurcumin	−5.30	130.44 *μ*M	Arg158, Ser276, Arg279	Leu283
Curcumin (keto)	−5.27	136.46 *μ*M	Arg158, Asn271, Arg279, Lys282	Ala274, Ala275, Leu283
Curcumin (enol)	−5.25	141.66 *μ*M	Arg158, Ser276, Lys282	Ala275, Leu283
CHC005∗	−5.24	144.93 *μ*M	Arg158	Leu283
CHC001∗	−5.19	156.49 *μ*M	Arg158, Lys282	Ala275, Leu283, Ile286
*α*-Turmerone	−5.13	172.61 *μ*M	Lys282	Ala150, Ala151, Leu283
*β*-Turmerone	−5.05	197.55 *μ*M	Lys282	Ala275, Leu283
Tetrahydrocurcumin	−5.05	199.62 *μ*M	Arg155, Arg158, Ser276, Arg279	Leu283
Curcumin glucuronide	−4.61	418.23 *μ*M	Arg155, Arg158, Arg279, Lys282	Ala151, Leu283, Ile286
Dihydroguaiaretic acid^*ψ*^	−4.43	569.58 *μ*M	Ser278, Arg279	Ala151, Ala275, Leu283
Resveratrol^*ψ*^	−4.20	829.30 *μ*M	Ser154, Lys282	Ala151, Leu283
Hexahydrocurcuminol	−4.08	1.02 mM	Arg155, Arg158, Ser276, Lys280, Lys282	Ala275, Leu283
Hexahydrocurcumin	−4.07	1.04 mM	Arg158, Ser276, Arg279	Ala275, Leu283, Ile286

*Synthetic curcumin-based inhibitors of Jun-Fos-DNA complex formation.

^*ψ*^Known inhibitors of Jun-Fos-DNA complex formation.
